# Saline Infusion in Cholera Cases

**Published:** 1909-12-11

**Authors:** 


### Tropical Medicine.

### SALINE INFUSION IN CHOLERA CASES.

In an article in the *Journal of the Royal Army Medical Corps*, upon the treatment of cholera, Captain S. A. Balck, V.H.S., R.A.M.C., and Lieutenant A. Harper Napier, I.M.S., record their personal experiences of a severe epidemic of cholera in India, and they make the very pertinent remark: "If we venture to dilate on the methods of treatment employed it is because we were to a large extent groping in the dark and feeling our way. The statements in the text-books are vague, and, while giving valuable indications, leave the details as to quantity and method of administration very largely undefined."

The cases they had to deal with presented no particular signs or symptoms other than those described in any clinical account of cholera. The only point upon which the text-books seem to lay too little stress is the characteristic odour of the stools: an odour difficult to describe—somewhat mousy, and at the same time slightly sour.

The treatment *par excellence* for cholera was found to be by saline injections, and of these the intravenous form is vastly preferable to the subcutaneous. The reaction to these is much quicker, and cholera is essentially a disease in which time is an object. The quantity of saline that can be administered intravenously is also greater than the amount that can be given under the skin. The authors we are quoting give it as their opinion that large quantities, not less than two quarts, should be given at each injection; that there should be no hesitation in repeating this at short intervals, even so short as half an hour sometimes; and that these repetitions should be persevered with even though the results appear at the time to be disappointing. One case in particular emphasised the importance of this. The patient, a young soldier, had been doing fairly well when his condition took a sudden turn for the worse, and the pulse began to fail. Four pints of saline were injected intravenously with marvellous improvement, which unfortunately was evanescent. Twenty minutes after the injection had been stopped the pulse was again hardly perceptible. The injection was repeated with the same result—temporary improvement followed by a rapid decline. The question of giving a third injection was debated and it was finally decided upon rather as a desperate remedy than from any real hope of its doing any good. This time, however, the pulse not only improved as before, but maintained its vigour; the patient never looked back, recovering completely.

The strength of the solution seems to be of little importance within certain limits. Our authors tried both the normal saline and the double-strength solution recommended by Rogers, and they cannot say that one acted appreciably better than the other. The method they employed was as follows: The vein selected—the median basilic as a rule—was cut down upon and ligatured, the ends of the ligature being left long. An opening for the cannula was made on the proximal side of the ligature, but the cannula was not tied in. On the conclusion of the injection the wound was dressed with a pad in the ordinary way, no further ligature being found necessary. Should it be needful to repeat the injection the long ends of the ligature are most useful in raising the vein from the tissues and helping to find the opening. One vein can be used for two or even three injections, after which the other arm may be requisitioned if need be. As a matter of fact, a clot was frequently found in the proximal end of the vein; so that a danger one has always to bear in mind is that such a clot may be dislodged into the general circulation with untoward results. For this reason it is perhaps safer not to use the same vein a second time unless the second injection follows the first very rapidly.

In desperate cases it was found of great service to give strychnine, not hypodermically, but by injecting the contents of the syringe into the funnel containing the saline. The strychnine thus entered the blood-stream directly, and five minims of the liquor could be distributed over two or three funnelfuls with excellent results.

Of other drugs very few proved of any use. Liquor hydrargyri perchloridi with or without opium was tried repeatedly but without any obvious benefit. Bismuth was equally ineffectual. Strychnine, ether and digitalin were of value in collapsed cases, either between the saline injections or reinforcing them. Adrenalin was not tried.

As regards individual symptoms the best thing for the distressing cramps is massage. Atropine was administered hypodermically at the same time, and may have had some part in lessening the pain of muscular contractions. Not one of the many remedies tried seemed to be able to check the vomiting; and the diarrhœa of the cholera-typhoid period also proved very little amenable to treatment. Starch and opium enemata, large astringent enemata, as well as the usual sedatives and astringents by the mouth, were all tried but without marked success. The most troublesome symptom, and one that proved fatal in several cases during the convalescent stage, was suppression of urine. Hot baths, fomentations, cupping, and diuretics were all in vain. The only method of treatment that was at all successful was to give half a grain of calomel at night and tincture of digitalis with potassium citrate four-hourly the next day. This old method of treatment of cardiac cases with failure of compensation and scanty urine seemed to do some good in more than one case of acute suppression of urine during cholera.

The diet consisted of beef tea, milk, and chicken broth alternately in two-hourly feeds of four ounces each. At first half an ounce of brandy was prescribed with each feed, but this was subsequently reduced. The patients themselves disliked the brandy. Champagne was tried instead, but it did not prove a success; the patients could with difficulty be induced to take it.

